# Endotracheal granulation after tracheostomy in acute respiratory distress syndrome from COVID‐19

**DOI:** 10.1002/rcr2.787

**Published:** 2021-05-26

**Authors:** Haruko Matsumoto, Shigeo Hanada, Kazumasa Yamamoto, Hisashi Takaya

**Affiliations:** ^1^ Department of Respiratory Medicine Toranomon Hospital Kajigaya Kawasaki Japan; ^2^ Okinaka Memorial Institute for Medical Research Tokyo Japan

**Keywords:** COVID‐19, endotracheal granulation, tracheostomy

## Abstract

Granulation tissue formation is one of the late complications of tracheostomy. It can cause stomal stenosis secondary to chondritis because of disproportionate excision of the anterior cartilage. Clinicians should carefully determine the incision point, which is typically located half way between the cricoid cartilage and the sternal notch.

## Graphical abstract

Tracheostomy requires an incision between the cricoid cartilage and sternal notch in critically ill patients who require an extended period of mechanical ventilation. This procedure can potentially increase the availability of intensive care unit beds in the coronavirus disease 2019 (COVID‐19) era. Granulation tissue formation can cause stomal stenosis secondary to chondritis because of the disproportionate excision of the anterior cartilage. Here, computed tomography (CT) imaging showed partial destruction of the cricoid cartilage, which is suggestive of chondritis and may lead to airway stenosis.

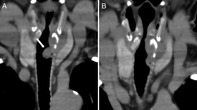

## Clinical Image

**Figure 1:**
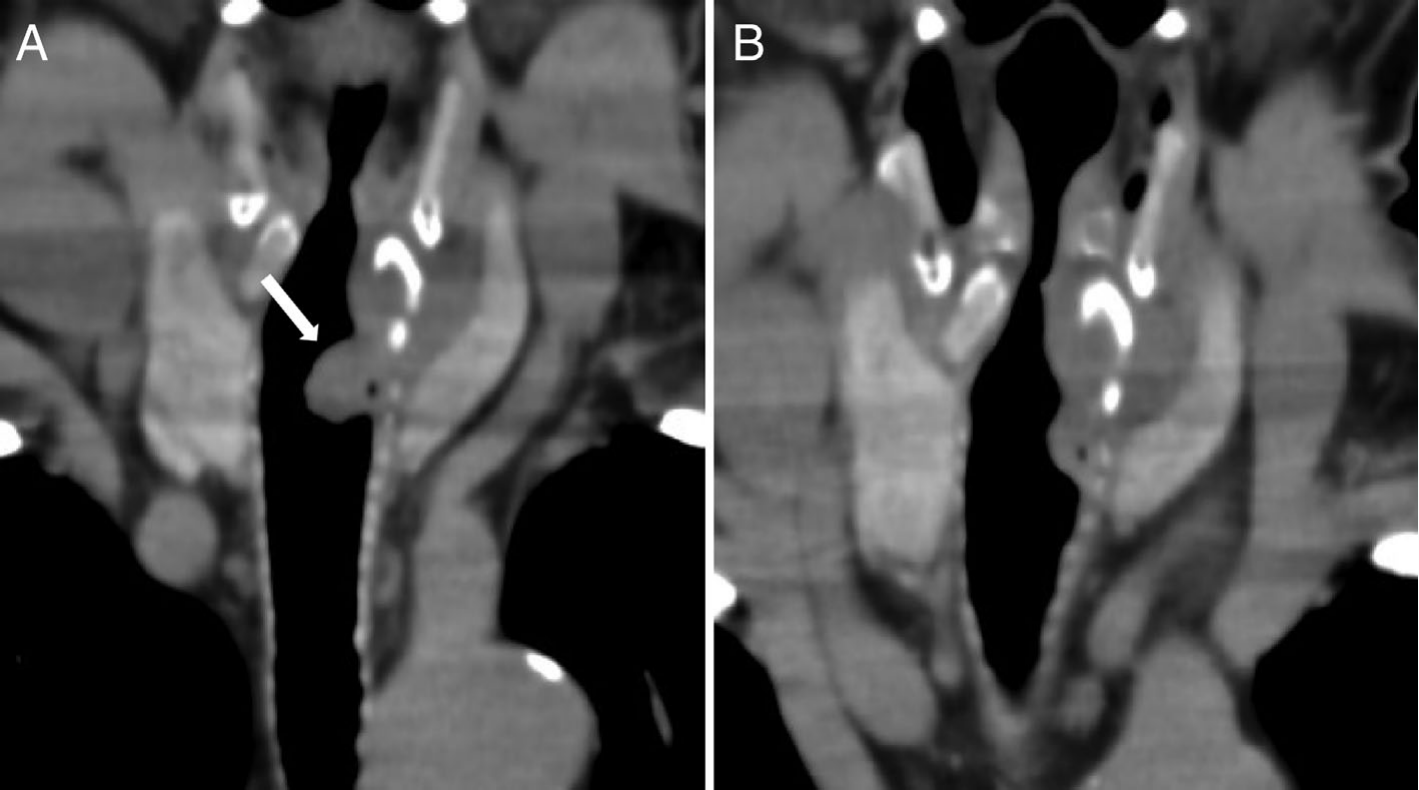
Computed tomography (CT) scan findings showing a solid elevated lesion with a 15‐mm diameter at the site of tracheostomy, and partial destruction of the left side of the cricoid cartilage with soft tissue thickening, suggesting cricoid chondritis (arrow) (A). CT scan findings showing an improvement of the elevated lesion after a four‐week treatment with a combination of inhaled glucocorticoid and two bronchodilators (long‐acting β2‐agonist plus a long‐acting muscarinic antagonist) (B).

**Figure 2:**
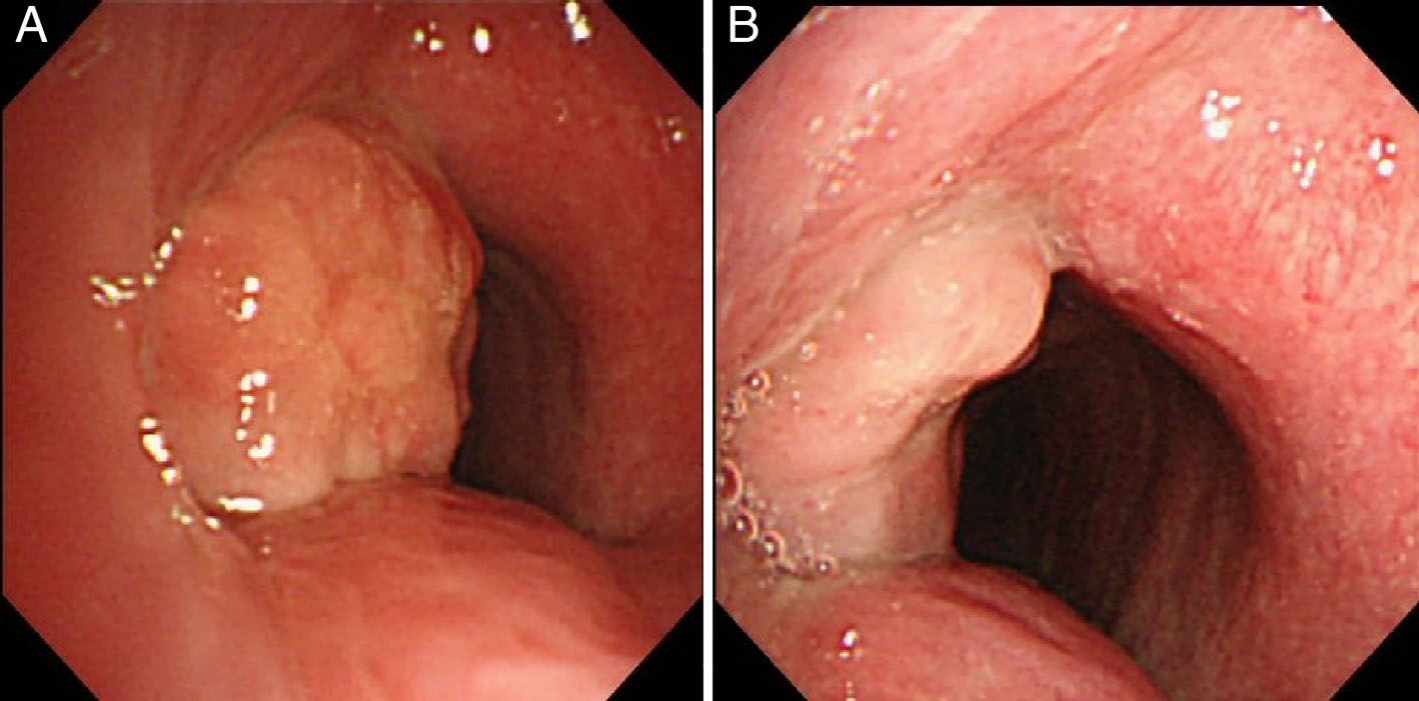
Fibre‐optic bronchoscopy imaging findings reveal a white elevated lesion arising from the left side of the trachea with significant narrowing of the airway (A). Bronchoscopic findings markedly improved after a four‐week treatment with combination of inhaled glucocorticoid and two bronchodilators (long‐acting β2‐agonist plus a long‐acting muscarinic antagonist) (B).

A 59‐year‐old man with acute respiratory distress syndrome (i.e. coronavirus disease (COVID‐19)) caused by severe acute respiratory syndrome coronavirus 2 (SARS‐CoV‐2) infection underwent percutaneous tracheostomy and mechanical ventilation for three weeks. He was readmitted six months after decannulation with dyspnoea, productive cough, and hoarseness. Computed tomography (CT) revealed a lesion protruding into the tracheal lumen at the tracheostomy site (Fig. 1A). Fibre‐optic bronchoscopy revealed stomal granulation tissue development in the subglottic airway (Fig. 2A). At readmission, his symptoms were mild and his oxygen saturation was 99% while he was breathing ambient air at rest. A treatment with inhaled glucocorticoid and two bronchodilators (long‐acting β2‐agonist plus a long‐acting muscarinic antagonist) was provided. His symptoms were carefully observed without performing an interventional bronchoscopic procedure and were improved at four weeks after treatment (Figs 1B, 2B). Tracheostomy requires an incision between the cricoid cartilage and sternal notch in critically ill patients who require an extended period of mechanical ventilation. This procedure can potentially increase the availability of intensive care unit beds in the COVID‐19 era [1]. Granulation tissue formation can cause stomal stenosis secondary to chondritis because of the disproportionate excision of the anterior cartilage [2]. Here, CT imaging showed partial destruction of the cricoid cartilage, which is suggestive of chondritis and may lead to airway stenosis. In case of suspicion of tracheal stenosis after imaging screening, bronchoscopy should be performed to define the exact site and cause of stenosis and the length of the involved trachea. A CT scan before decannulation or immediately after decannulation before closing of the tracheostomy is the preferred way to assess airway stenosis based on the strategies to minimize aerosol generation by bronchoscopy.

### Disclosure Statement

Appropriate written informed consent was obtained for publication of this case report and accompanying images.

### Author Contribution Statement

All authors directly provided patient care. Haruko Matsumoto and Shigeo Hanada drafted and edited the manuscript and images. All authors revised the manuscript.
